# Enhancing Patient Safety: Audit of Medicines Reconciliation of Psychiatry Inpatients in NHS Lanarkshire 2023–24

**DOI:** 10.1192/bjo.2025.10609

**Published:** 2025-06-20

**Authors:** Arya Iyer

**Affiliations:** NHS Lanarkshire, Glasgow, United Kingdom

## Abstract

**Aims:** Medication errors at the interface of care (admission, transfer and discharge) are a leading cause of patient morbidity and mortality. For this reason, the Scottish Patient Safety Programme and National Institute for Health and Care Excellence (NICE) have highlighted the need for accurate medicines reconciliation, and set a 95% standard that all medicines should be reconciled within 24 hours of the patient’s admission. This audit intended to assess quality of completion of Medicines Reconciliation forms and identify any potential barriers to completion. The objectives of this audit were to assess current adherence to local Medicines Reconciliation guidelines across General Adult Psychiatry Wards 19 and 20 in University Hospital Hairmyres (UHH) and identify any potential factors which may be contributing to Medicines Reconciliation forms not being completed appropriately.

**Methods:** An audit of Medicines Reconciliation form completion for admissions to Wards 19 and 20 in UHH was carried out retrospectively for all (24 no.) patients admitted from 10/11/2023–12/12/2023 using electronic case notes. Other systems, including the software for online prescribing and TrakCare were also used. Each section of the proforma was assessed and information recorded in an Excel spreadsheet as well as information about whether this was completed in the first 24 hours of admission. Following this, a summary document with the latest guidelines and the link to an e-learning module were distributed amongst the Resident Doctors, and raised at the monthly Resident Doctor’s meeting. The form completion was then re-audited for patients admitted from 11/3/24–11/4/24 (34 no.).

**Results:** In the first cycle of the audit, only 70% of patients had their form completed within 24 hours of admission, which then improved to 100% in the second cycle. Another section with poor compliance in the first cycle was the section confirming that 2 sources of information had been used (66% completed), which also increased to 100% in the re-audit. In terms of the other parameters assessed, there were improvements in all 12 areas.

**Conclusion:** The audit was straightforward to carry out and yielded valuable insights to improving inpatient psychiatry care. However, the results may have been influenced by individual variability, given the involvement of multiple doctors in the clerking process for new admissions. The findings highlight the importance of regular training and reinforcement of local guidelines to enhance patient care. Nonetheless, there remains an opportunity for further work in this area, including regional audits and updates to local guidelines.

